# Supplementary material for: Levels and determinants of person-centred maternity care among women living in urban informal settlements: evidence from client exit surveys in Nairobi, Lusaka and Ouagadougou
Source: BMJ Glob Health. 2025 Mar 15;10(3):e017337. doi: 10.1136/bmjgh-2024-017337 (PMC11911685; doi:10.1136/bmjgh-2024-017337)
Supplement: online supplemental file 1 [file bmjgh-10-3-s001.pdf]

Levels And Determinants of Person-Centered Maternity Care Among Women Living in Urban Informal Settlements: Evidence from Client Exit Surveys in Nairobi, Lusaka and Ouagadougou.

SUPPLEMENTAL MATERIALS

Supplemental table 1. PCMC scale questions, response categories and scoring

Question	Response categories	PCMC scoring (out of 90)
**DIGNITY & RESPECT**		
Did the doctors, nurses, or other staff at the facility treat you with respect?	No, never	0
	Yes, a few times	1
	Yes, most of the time	2
	Yes, all the time	3
Did the doctors, nurses, or other staff at the facility treat you in a friendly manner?	No, never	0
	Yes, a few times	1
	Yes, most of the time	2
	Yes, all the time	3
Did you feel that they shouted at you, scolded, insulted, threatened, or talked to you rudely?	No, never	3
	Yes, once	2
	Yes, a few times	1
	Yes, many times	0
	Refused to respond	3
Did you feel like you were treated roughly like pushed, beaten, slapped, pinched, physically restrained, or gagged?	No, never	3
	Yes, once	2
	Yes, a few times	1
	Yes, many times	0
	Refused to respond	3
During examinations in the labor room, were you covered up with a cloth or blanket, or screened with a curtain so that you did not feel exposed?	No, never	0
	Yes, a few times	1
	Yes, most of the time	2
	Yes, all the time	3
Do you feel like your health information was or will be kept confidential at this facility?	No, never	0
	Yes, a few times	1
	Yes, most of the time	2
	Yes, all the time	3
**COMMUNICATION & AUTONOMY**		
During your time in the health facility did the doctors, nurses, or other health-care providers introduce themselves to you when they first came to see you?	No, none of them	0
	Yes, a few of them	1
	Yes, most of them	2
	Yes, all of them	3

Did the doctors, nurses, or other health-care providers call you by your name?	No, never	0
	Yes, a few times	1
	Yes, most of the time	2
	Yes, all the time	3
Did you feel like the doctors, nurses or other staff at the facility involved you in decisions about your care?	No, never	0
	Yes, a few times	1
	Yes, most of the time	2
	Yes, all the time	3
	Did not have to make any decisions	3
During the delivery, do you feel like you were able to be in the position of your choice?	No, never	0
	Yes, for a short time	1
	Yes, most of the time	2
	Yes, all the time	3
Did the doctors, nurses, or other staff at the facility speak to you in a language you could understand?	No, never	0
	Yes, a few times	1
	Yes, most of the time	2
	Yes, all the time	3
Did the doctors, nurses, or other staff at the facility ask your permission or consent before doing procedures on you?	No, never	0
	Yes, a few times	1
	Yes, most of the time	2
	Yes, all the time	3
Did the doctors, nurses, or other staff at the facility explain to you why they were doing examinations or procedures on you?	No, never	0
	Yes, a few times	1
	Yes, most of the time	2
	Yes, all the time	3
Did the doctors, nurses, or other staff at the facility explain to you why they were giving you any medicine?	No, never	0
	Yes, a few times	1
	Yes, most of the time	2
	Yes, all the time	3
	Did not get any medicine	3
Did you feel you could ask the doctors, nurses, or other staff at the facility any questions you had?	No, never	0
	Yes, a few times	1
	Yes, most of the time	2
	Yes, all the time	3
**SUPPORTIVE CARE**		
How did you feel about the amount of time you waited to receive care? Would you say it was:	Very short	3
	Somewhat short	2
	Somewhat long	1
	Very long	0
Did the doctors and nurses at the facility show concern for your feelings about your delivery?	No, never	0
	Yes, a few times	1
	Yes, most of the time	2

	Yes, all the time	3
Did the doctors, nurses, or other staff at the facility try to understand your anxieties?	No, never	0
	Yes, a few times	1
	Yes, most of the time	2
	Yes, all the time	3
	Did not have any anxiety	3
When you needed help, did you feel the doctors, nurses, or other staff at the facility paid attention?	No, never	0
	Yes, a few times	1
	Yes, most of the time	2
	Yes, all the time	3
Do you feel the doctors or nurses did everything they could to help control your pain?	No, never	0
	Yes, a few times	1
	Yes, most of the time	2
	Yes, all the time	3
Were you allowed to have someone you wanted (outside of staff at the facility, such as family or friends) to stay with you during labor?	No, never	0
	Yes, a few times	1
	Yes, most of the time	2
	Yes, all the time	3
	I did not want someone to stay with me	3
Were you allowed to have someone you wanted to stay with you during delivery?	No, never	0
	Yes, a few times	1
	Yes, most of the time	2
	Yes, all the time	3
	I did not want someone to stay with me	3
Did you feel the doctors, nurses, or other staff at the facility took good care of you, at the best of their ability?	No, never	0
	Yes, a few times	1
	Yes, most of the time	2
	Yes, all the time	3
Did you feel you could completely trust the doctors, nurses, or other staff at the facility with regards to your care?	No, never	0
	Yes, a few times	1
	Yes, most of the time	2
	Yes, all the time	3
Do you think there were enough health staff in the facility to care for you?	No, never	0
	Yes, a few times	1
	Yes, most of the time	2
	Yes, all the time	3
Thinking about the labor and postnatal wards, did you feel the health facility was crowded?	No, never	3
	Yes, a few times	2
	Yes, most of the time	1
	Yes, all the time	0
	Very dirty	0

Thinking about the wards, washrooms, and the general environment of the health facility, would you say the facility was very clean, clean, dirty, or very dirty?	Dirty	1
	Clean	2
	Very clean	3
Was there running water in the facility?	No, never	0
	Yes, a few times	1
	Yes, most of the time	2
	Yes, all the time	3
Was there electricity in the facility?	No, never	0
	Yes, a few times	1
	Yes, most of the time	2
	Yes, all the time	3
In general, did you feel safe in the health facility?	No, never	0
	Yes, a few times	1
	Yes, most of the time	2
	Yes, all the time	3

Supplemental figure 1. Study conceptual framework

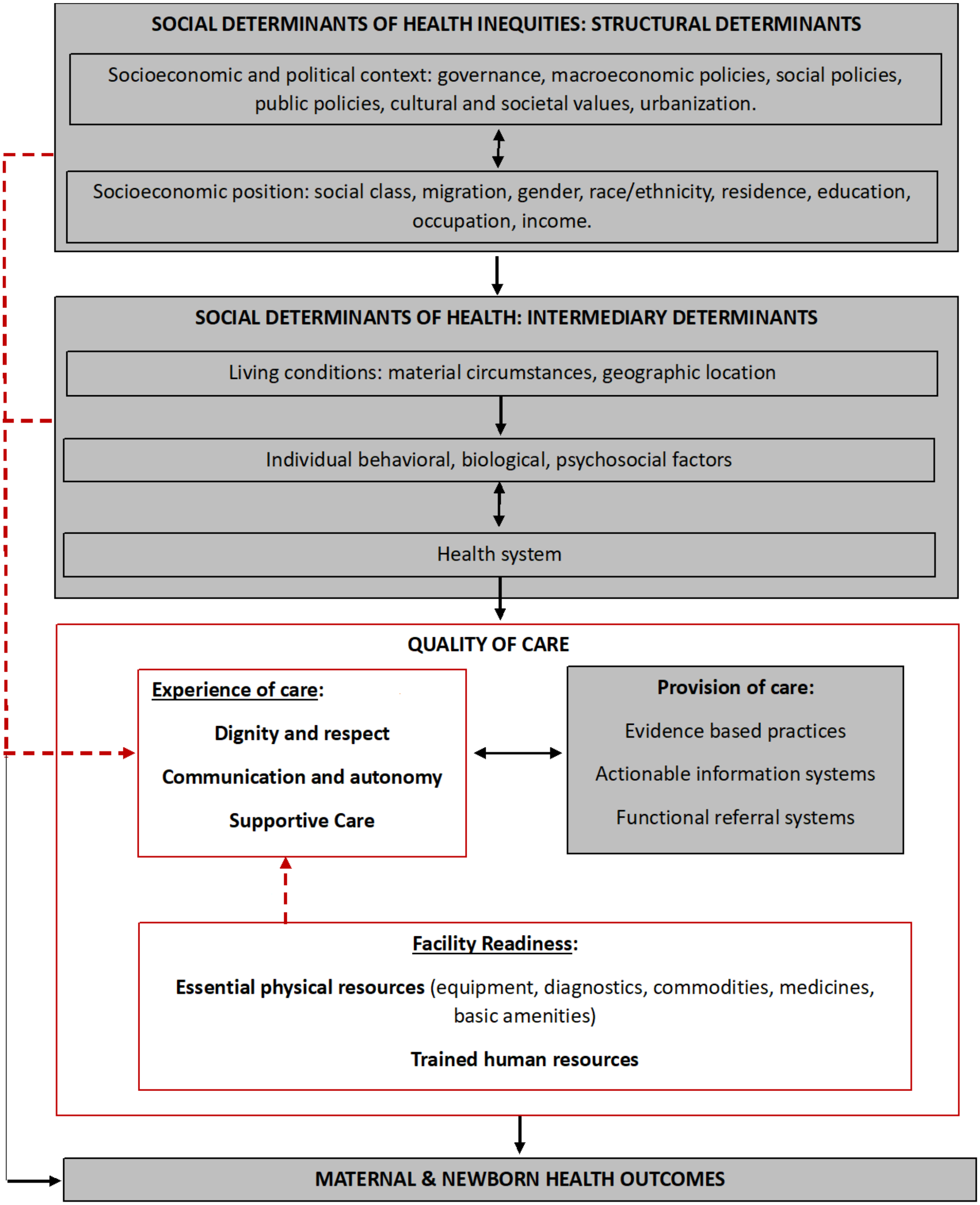

Supplemental figure 2. Histogram and Q-Q plot of unscaled PCMC score (out of 90) by study site

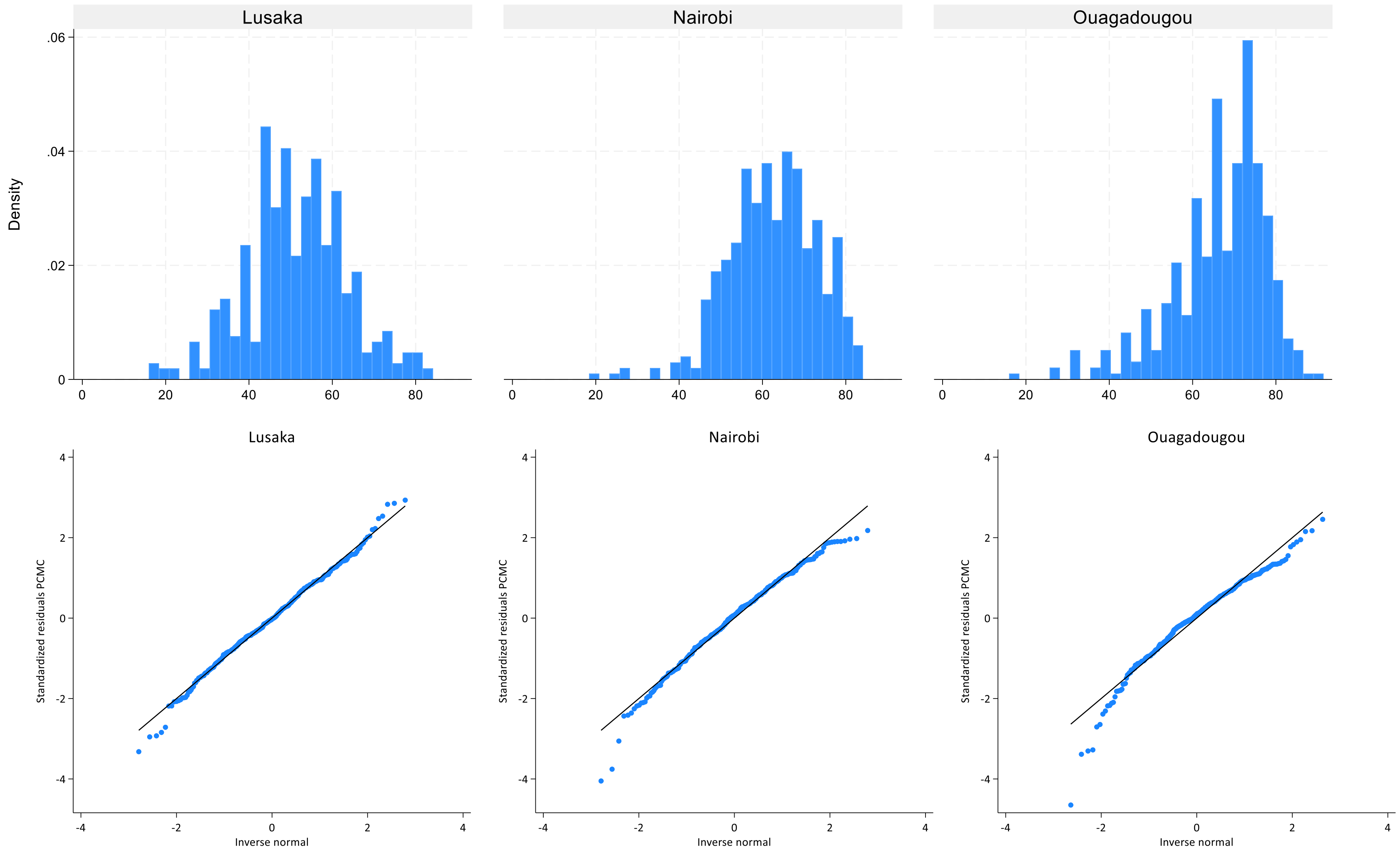

Supplemental table 2. Sensitivity analysis: PCMC logistic regression by study site.

	LUSAKA Odds Ratio	NAIROBI Odds Ratio	OUAGADOUGOU Odds Ratio
STRUCTURAL DETERMINANTS			
**Education** (ref: primary or less)			
Secondary or more	1.12 [0.60,2.10]	0.99 [0.58,1.69]	0.88 [0.37,2.09]
**Employment** (ref: unemployed)			
Employed (public, private, self)	0.91 [0.50,1.63]	1.13 [0.61,2.08]	**5.08*** **[1.47,17.49]**
Informal/casual labor	1.68 [0.62,4.55]	1.00 [0.48,2.08]	1.10 [0.48,2.48]
INTERMEDIARY DETERMINANTS			
**Age** (ref: 20-35 yrs)			
15-19 yrs	0.44 [0.16,1.25]	0.93 [0.33,2.65]	1.16 [0.24,5.67]
35-49 yrs	1.21 [0.46,3.19]	1.66 [0.71,3.87]	1.22 [0.34,4.34]
**Marital status** (ref: in union)			
Not in union	**2.27*** **[1.09,4.72]**	0.86 [0.42,1.76]	0.74 [0.06,9.46]
**Parity** (ref: 2-3 children)			
1	1.15 [0.56,2.38]	0.64 [0.34,1.19]	0.90 [0.32,2.55]
4+	0.86 [0.38,1.94]	0.67 [0.30,1.49]	0.67 [0.25,1.83]
**Pregnancy complications** (ref: No)			
Yes	0.63 [0.21,1.89]	1.55 [0.73,3.30]	1.25 [0.31,5.00]
**Miscarriage/Stillbirth history** (ref: No)			
Yes	0.65 [0.30,1.39]	0.73 [0.37,1.45]	1.65 [0.69,3.98]
**Number of ANC contacts** (ref: 1-3)			
0	1.21 [0.16,9.05]	0.13 [0.01,1.60]	4.20 [0.25,70.69]
4+	0.91 [0.49,1.69]	1.39 [0.80,2.43]	1.19 [0.54,2.63]
**Place of ANC** (ref: Different facility/home/no ANC)			
Same facility as place of delivery	0.87 [0.49,1.56]	0.82 [0.45,1.50]	0.92 [0.39,2.17]
HEALTH SYSTEMS DETERMINANTS			
**Delivery facility type** (ref: Health center)			
Hospital	1.20 [0.25,5.83]	**0.24**** **[0.12,0.49]**	1.30 [0.26,6.52]
**Delivery facility managing authority/ownership** (ref: Public)			
Private for profit	**-**	1.99	6.50

		[0.55,7.12]	[0.50,84.75]
Private non-profit/faith-based	-	1.75	2.01
		[0.64,4.78]	[0.38,10.49]
**Assistance during delivery** (ref: midwife /nurse /TBA)			
Physician/specialist	1.67	**2.62****	0.27
	[0.70,3.99]	**[1.55,4.43]**	[0.03,2.77]
Other/unskilled	1.27	-	-
	[0.15,10.49]		
Don't Know/Couldn't distinguish	0.29	2.15	0.48
	[0.03,2.90]	[0.42,10.98]	[0.18,1.27]
**Maternal PNC before discharge** (ref: No)			
Yes	-	0.68	0.41
		[0.12,3.83]	[0.07,2.49]
**Length of facility stay** (ref: <24h)			
≥24h	0.54	0.98	**0.32***
	[0.28,1.02]	[0.47,2.07]	**[0.13,0.77]**
**PNC counseling: danger signs** (ref: No)			
Yes	**2.44***	**3.81****	2.57
	**[1.19,4.98]**	**[2.17,6.69]**	[0.94,7.02]
**PNC counselling: family planning** (ref: No)			
Yes	0.81	0.86	0.88
	[0.44,1.49]	[0.50,1.48]	[0.34,2.27]
**PNC: BP check** (ref: No)			
Yes	4.09	0.96	1.46
	[0.43,38.48]	[0.29,3.11]	[0.38,5.59]
**PNC: newborn check** (ref: No)			
Yes	-	**8.33****	**8.22****
		**[2.35,29.62]**	**[1.77,38.29]**
**PNC: newborn appointment** (ref: No)			
Yes	0.59	0.71	4.01
	[0.10,3.53]	[0.19,2.69]	[0.64,24.92]
Observations (n)	377	405	369

95% confidence intervals in brackets

* $p < 0.05$, ** $p < 0.01$

Supplemental table 3. Care seeking and content of antenatal care during pregnancy by study site.

	Lusaka (n=436)		Nairobi (n=412)		Ouagadougou (n=401)	
	%	[95%CI]	%	[95%CI]	%	[95%CI]
**Place of ANC**						
Home/no ANC	1.8	[0.9-3.6]	1.0	[0.4-2.6]	2.5	[1.3-4.6]
Same health facility as place of delivery	62.8	[58.2-67.3]	33.2	[28.9-38.0]	71.1	[66.4-75.3]
Other health facility	35.3	[31.0-39.9]	65.8	[61.0-70.2]	26.4	[22.3-31.0]
**Place of ANC: facility type**						
Home/no ANC	1.8	[0.9-3.6]	1.0	[0.4-2.6]	2.5	[1.3-4.6]
Hospital	51.8	[47.1-56.5]	24.3	[20.4-28.7]	26.9	[22.8-31.5]
Health center/other	46.3	[41.6-51.0]	74.8	[70.3-78.7]	70.6	[65.9-74.8]
**Number of ANC contacts**						
No ANC	1.4	[0.6-3.0]	1.0	[0.4-2.6]	2.5	[1.3-4.6]
1-3 contacts	30.0	[25.9-34.5]	32.5	[28.2-37.2]	27.2	[23.0-31.8]
4-7 contacts	61.9	[57.3-66.4]	62.9	[58.1-67.4]	63.8	[59.0-68.4]
8+ contacts	6.7	[4.7-9.4]	3.4	[2.0-5.7]	1.2	[0.5-3.0]
**Timely ANC initiation (first trimester)**	22.3	[18.6-26.5]	23.8	[19.9-28.2]	31.7	[27.3-36.5]
**ANC content**						
Blood pressure	99.1	[97.5-99.7]	99.8	[98.3-100.0]	97.7	[95.6-98.8]
Blood sample	91.4	[88.3-93.7]	98.8	[97.1-99.5]	92.6	[89.5-94.8]
Urine sample	72.1	[67.6-76.1]	98.5	[96.8-99.3]	64.5	[59.6-69.1]
HIV test	97.9	[96.0-98.9]	99.5	[98.1-99.9]	74.7	[70.1-78.8]
Counseling: pregnancy danger signs	88.6	[85.2-91.3]	88.5	[85.0-91.2]	69.6	[64.8-73.9]
Counseling: nutrition	94.0	[91.3-95.9]	86.5	[82.8-89.5]	52.2	[47.2-57.1]
Any IFA Supplementation	94.4	[91.8-96.2]	76.7	[72.3-80.6]	98.2	[96.3-99.1]
Any IPTp (SP/Fansidar)	95.1	[92.6-96.8]	31.9	[27.5-36.6]	97.7	[95.6-98.8]
Tetanus toxoid vaccine					83.9	[79.9-87.2]
**ANC content score* (median, IQR)**	100	[87.5-100]	87.5	[75.0- 87.5]	77.8	[66.7-88.9]

**ANC score defined as the number of ANC content interventions received, out of the number of interventions collected, expressed as a percentage.*

Supplemental figure 3: Distribution of overall PCMC scores (%) by study site

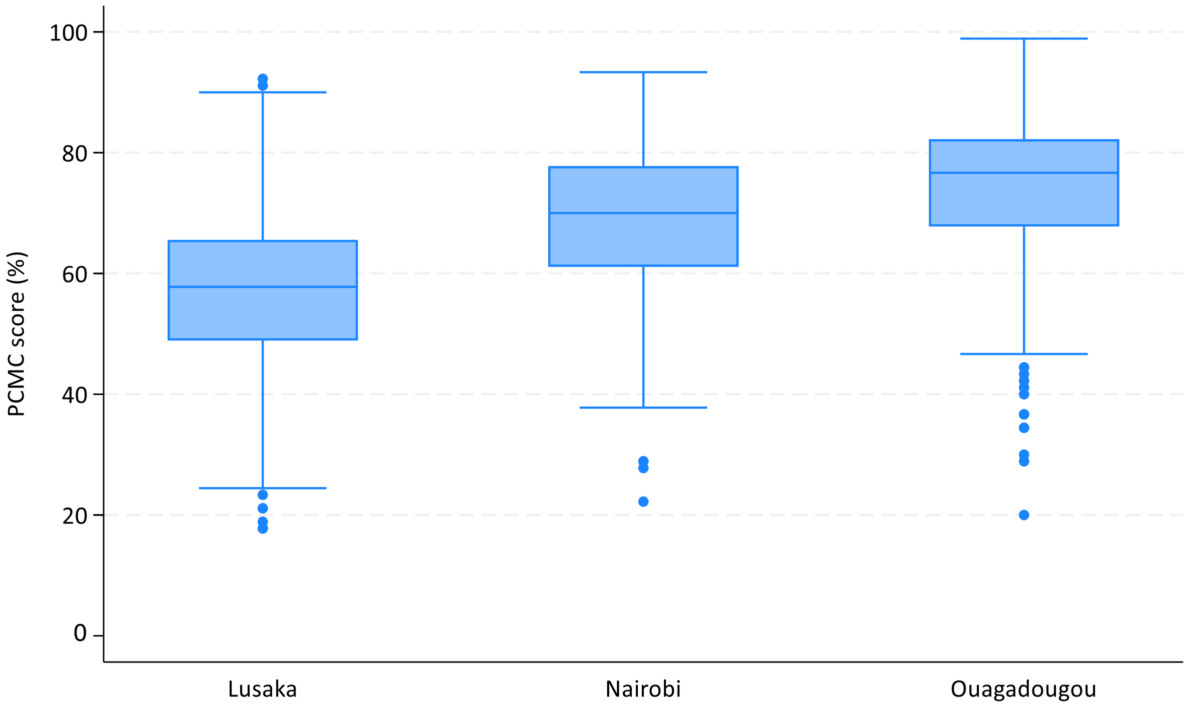

Supplemental figures 4 A-B. PCMC item responses (%) for dignity and respect (A), and supportive care (B) domains by study site.

A. Dignity and respect

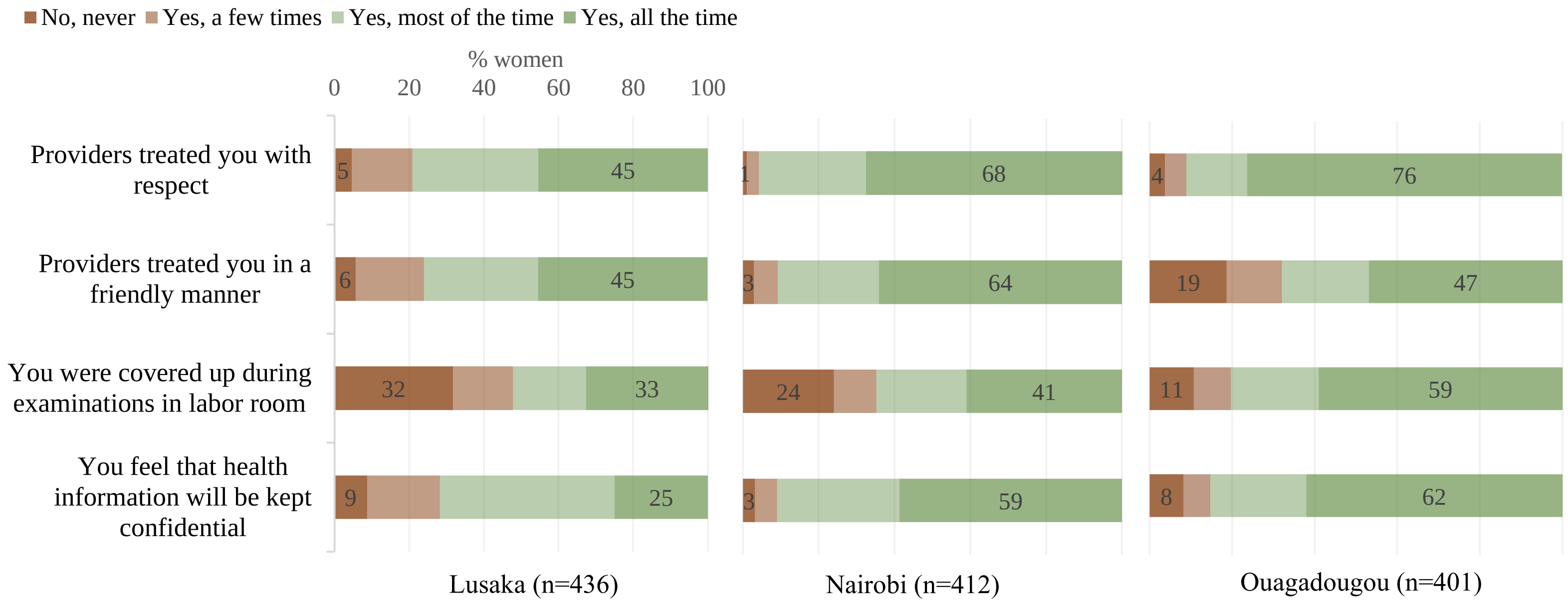

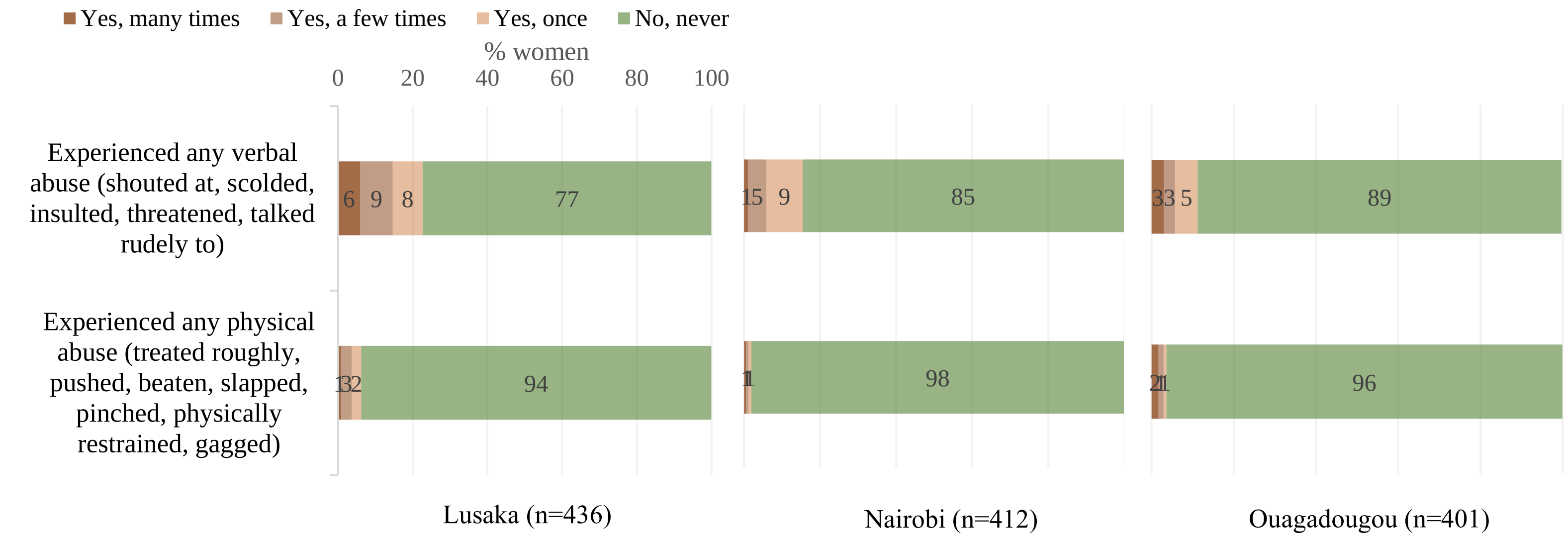

B. Supportive care

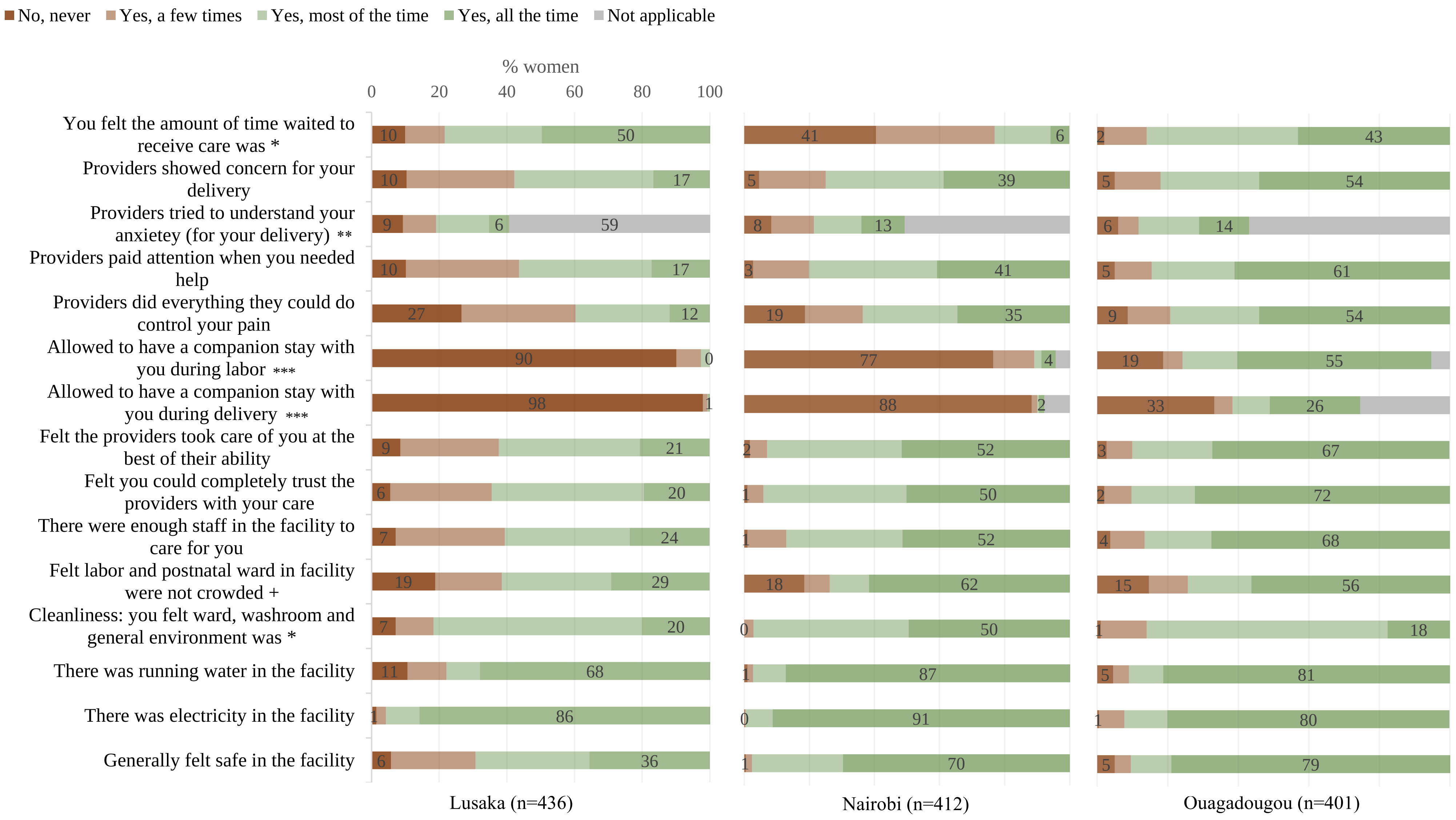

**response categories differ:*
amount of time waited: very long (dark red), somewhat long, somewhat short, very short (dark green);
cleanliness: very dirty (dark red), dirty, clean, very clean (dark green).
*** Not applicable response: "I was not feeling anxious".*
**** Not applicable response: "I did not want someone to stay with me".*
+ crowdedness: crowded all the time (dark red), most of the time, a few times, never crowded (dark green).

Supplemental tables 5 A-C. Structural, intermediary and health systems determinants of overall PCMC by study site.

A. Lusaka: Structural, intermediary, health systems determinants of PCMC (unscaled score, out of 90)

	Bivariate	Model 1	Model 2	Model 3
	STRUCTURAL DETERMINANTS			
**Education** (ref: primary or less)				
Secondary or more	0.30 [-2.01,2.61]	0.17 [-2.13,2.48]	0.31 [-2.10,2.71]	-0.89 [-3.18,1.40]
**Employment** (ref: unemployed)				
Employed: public, private, self	**2.66*** **[0.40,4.91]**	**2.65*** **[0.39,4.91]**	**2.49*** **[0.18,4.80]**	1.33 [-0.89,3.55]
Informal/casual labor	0.25 [-3.58,4.07]	0.27 [-3.57,4.10]	-0.04 [-3.91,3.84]	-0.98 [-4.65,2.69]
	INTERMEDIARY DETERMINANTS			
**Age** (ref: 20-35 yrs)				
15-19 yrs	-2.23 [-5.56,1.10]		-0.87 [-4.70,2.97]	-1.50 [-5.09,2.09]
35-49 yrs	-0.75 [-4.01,2.50]		-0.24 [-4.10,3.62]	-0.67 [-4.36,3.03]
**Marital status** (ref: in union)				
Not in union	0.24 [-2.35,2.83]		1.32 [-1.58,4.22]	1.79 [-0.96,4.53]
**Parity** (ref: 2-3 children)				
1	-1.76 [-4.22,0.71]		-1.97 [-4.87,0.92]	-0.61 [-3.41,2.18]
4+	-0.93 [-3.57,1.72]		-0.80 [-3.93,2.33]	-0.24 [-3.21,2.73]
**Pregnancy complications** (ref: No)				
Yes	0.67 [-2.98,4.32]		0.33 [-3.32,3.97]	-0.81 [-4.32,2.71]
**Miscarriage/Stillbirth history** (ref: No)				
Yes	-1.41 [-4.20,1.39]		-1.82 [-4.68,1.04]	-0.73 [-3.46,2.00]
**Number of ANC contacts** (ref: 1-3)				
0	0.63 [-8.54,9.81]		-0.33 [-9.61,8.94]	-0.44 [-9.12,8.24]
4+	0.39 [-1.93,2.71]		0.31 [-2.09,2.72]	0.57 [-1.73,2.87]
**Place of ANC** (ref: Different facility/home/no ANC)				
Same facility as place of delivery	-0.56 [-2.78,1.66]		-0.53 [-2.78,1.73]	-1.50 [-3.71,0.71]
	HEALTH SYSTEMS DETERMINANTS			
**Delivery facility type** (ref: Health				

center)				
Hospital	-0.92			-1.20
	[-8.00,6.17]			[-7.52,5.13]
**Assistance during delivery** (ref: midwife/nurse/TBA)				
Physician/specialist	2.43			**4.04***
	[-1.06,5.92]			**[0.62,7.46]**
Other/unskilled	-1.62			-1.75
	[-11.45,8.21]			[-10.91,7.42]
Don't Know/ Could not	-4.28			-3.10
distinguish	[-10.64,2.08]			[-9.16,2.97]
**Maternal PNC before discharge** (ref: No)				
Yes	**10.73****			1.79
	**[6.11,15.35]**			[-5.33,8.91]
**Length of facility stay** (ref: <24h)				
≥24h	0.08			-1.57
	[-2.31,2.47]			[-3.91,0.77]
**PNC counseling: danger signs** (ref: No)				
Yes	**7.81****			**6.74****
	**[5.63,9.98]**			**[4.20,9.28]**
**PNC counselling: family planning** (ref: No)				
Yes	**3.12****			-0.88
	**[0.99,5.26]**			[-3.22,1.45]
**PNC: BP check** (ref: No)				
Yes	**8.11****			2.59
	**[4.50,11.72]**			[-2.95,8.13]
**PNC: newborn check** (ref: No)				
Yes	**9.46****			**4.63***
	**[5.51,13.42]**			**[0.20,9.06]**
**PNC: newborn appointment** (ref: No)				
Yes	3.77			-0.09
	[-2.08,9.61]			[-5.71,5.54]
Observations (n)		436	434	419
AIC		3358.3	3358.6	3200.3

95% confidence intervals in brackets
* $p < 0.05$, ** $p < 0.01$

B. Nairobi: Structural, intermediary, health systems determinants of PCMC (unscaled score, out of 90)

	Bivariate	Model 1	Model 2	Model 3
STRUCTURAL DETERMINANTS				
**Education** (ref: primary or less)				
Secondary or more	-1.04 [-2.97,0.88]	-1.08 [-3.01,0.86]	-0.86 [-2.84,1.12]	-0.33 [-2.14,1.49]
**Employment** (ref: unemployed)				
Employed: public, private, self	0.22 [-1.93,2.37]	0.27 [-1.88,2.42]	0.07 [-2.09,2.24]	0.39 [-1.61,2.38]
Informal/casual labor	-0.23 [-2.88,2.41]	-0.33 [-2.97,2.32]	-0.11 [-2.76,2.54]	0.33 [-2.13,2.80]
INTERMEDIARY DETERMINANTS				
**Age** (ref: 20-35 yrs)				
15-19 yrs	-0.15 [-3.82,3.52]		-0.59 [-4.55,3.37]	-0.75 [-4.34,2.85]
35-49 yrs	1.96 [-0.86,4.78]		1.53 [-1.47,4.53]	1.83 [-0.95,4.61]
**Marital status** (ref: in union)				
Not in union	**-2.89*** **[-5.54,-0.25]**		-2.56 [-5.29,0.18]	**-2.50*** **[-4.99,-0.01]**
**Parity** (ref: 2-3 children)				
1	-1.08 [-3.09,0.92]		-0.68 [-2.97,1.60]	-1.28 [-3.40,0.84]
4+	0.25 [-2.64,3.13]		-0.07 [-3.04,2.90]	0.55 [-2.22,3.33]
**Pregnancy complications** (ref: No)				
Yes	0.17 [-2.36,2.69]		0.00 [-2.54,2.55]	0.67 [-1.69,3.04]
**Miscarriage/Stillbirth history** (ref: No)				
Yes	-1.00 [-3.39,1.39]		-1.47 [-3.91,0.97]	-1.24 [-3.52,1.04]
**Number of ANC contacts** (ref: 1-3)				
0	1.30 [-7.78,10.37]		1.28 [-7.91,10.48]	-0.87 [-9.31,7.57]
4+	0.39 [-1.53,2.30]		0.77 [-1.24,2.79]	1.23 [-0.61,3.08]
**Place of ANC** (ref: Different facility/home/no ANC)				
Same facility as place of delivery	0.56 [-1.65,2.76]		0.39 [-1.80,2.59]	-0.44 [-2.45,1.56]
HEALTH SYSTEMS DETERMINANTS				
**Delivery facility type** (ref: Health center)				
Hospital	**-9.48****			**-7.15****

	**[-13.11,-5.85]**			**[-10.42,-3.89]**
**Delivery facility managing authority/ownership** (ref: Public)				
Private for profit	5.39			2.59
	[-1.39,12.16]			[-1.78,6.97]
Private non-profit/faith-based	5.36			1.06
	[-0.56,11.28]			[-2.53,4.64]
**Assistance during delivery** (ref: midwife/nurse/TBA)				
Physician/specialist	**4.39****			**4.77****
	**[2.44,6.33]**			**[2.96,6.59]**
Don't Know/Could not distinguish	1.71			1.96
	[-4.61,8.02]			[-3.97,7.88]
**Maternal PNC before discharge** (ref: No)				
Yes	**7.90****			3.14
	**[3.49,12.31]**			[-2.21,8.50]
**Length of facility stay** (ref: <24h)				
≥24h	-0.69			-0.74
	[-3.23,1.86]			[-3.08,1.61]
**PNC counseling: danger signs** (ref: No)				
Yes	**5.50****			**4.74****
	**[3.63,7.37]**			**[2.80,6.68]**
**PNC counselling: family planning** (ref: No)				
Yes	**2.01***			-0.85
	**[0.03,3.98]**			[-2.76,1.07]
**PNC: BP check** (ref: No)				
Yes	**4.07****			2.27
	**[1.30,6.83]**			[-0.90,5.44]
**PNC: newborn check** (ref: No)				
Yes	**9.31****			**7.63****
	**[5.54,13.08]**			**[3.80,11.45]**
**PNC: newborn appointment** (ref: No)				
Yes	4.28			2.01
	[-0.34,8.90]			[-2.58,6.61]
Observations (n)		412	410	405
AIC		3024.0	3017.6	2909.9

95% confidence intervals in brackets

* $p < 0.05$, ** $p < 0.01$

C. Ouagadougou: Structural, intermediary, health systems determinants of PCMC (unscaled score, out of 90)

	Bivariate	Model 1	Model 2	Model 3
	STRUCTURAL DETERMINANTS			
**Education** (ref: primary or less)				
Secondary or more	0.69	0.15	0.35	0.41
	[-1.49,2.87]	[-2.06,2.37]	[-2.14,2.83]	[-1.82,2.65]
**Employment** (ref: unemployed)				
Employed (public, private,	**4.20****	**4.17****	**4.59****	**3.45***
self)	**[1.27,7.13]**	**[1.19,7.14]**	**[1.33,7.86]**	**[0.49,6.41]**
Informal/casual labor	0.77	0.79	1.01	0.87
	[-1.54,3.08]	[-1.54,3.12]	[-1.44,3.46]	[-1.34,3.07]
	INTERMEDIARY DETERMINANTS			
**Age** (ref: 20-35 yrs)				
15-19 yrs	1.22		2.77	2.47
	[-2.88,5.32]		[-1.78,7.32]	[-1.68,6.62]
35-49 yrs	2.23		3.20	1.41
	[-0.77,5.23]		[-0.42,6.81]	[-1.87,4.68]
**Marital status** (ref: in union)				
Not in union	-3.12		-0.63	0.54
	[-11.59,5.36]		[-9.22,7.97]	[-7.30,8.38]
**Parity** (ref: 2-3 children)				
1	-1.35		-1.59	-1.25
	[-4.05,1.35]		[-4.71,1.52]	[-4.05,1.56]
4+	-0.14		-1.31	-0.75
	[-2.55,2.26]		[-4.30,1.67]	[-3.43,1.94]
**Pregnancy complications** (ref: No)				
Yes	0.50		0.39	-0.43
	[-3.40,4.40]		[-3.57,4.35]	[-4.09,3.23]
**Miscarriage/Stillbirth history** (ref: No)				
Yes	1.21		1.41	1.16
	[-1.17,3.59]		[-1.13,3.95]	[-1.15,3.46]
**Number of ANC contacts** (ref: 1-3)				
0§	**7.33***		7.10	5.24
	**[0.57,14.09]**		[-0.14,14.34]	[-1.25,11.72]
4+	**2.43***		2.12	1.66
	**[0.13,4.74]**		[-0.25,4.50]	[-0.48,3.81]
**Place of ANC** (ref: Different facility/home/ no ANC)				
Same facility as place of	-0.20		0.50	1.00
delivery	[-2.55,2.14]		[-2.05,3.06]	[-1.34,3.34]
			-	-
	HEATH SYSTEMS DETERMINANTS			
**Delivery facility type** (ref: Health center)				
Hospital	3.40			0.41

	[-0.87,7.67]			[-3.94,4.77]
**Delivery facility managing authority/ownership** (ref: Public)				
Private for profit	**8.99****			**7.36***
	**[3.20,14.77]**			**[1.29,13.44]**
Private non-profit/faith-based	**5.68****			3.01
	**[1.68,9.69]**			[-1.39,7.42]
**Assistance during delivery** (ref: midwife/nurse/TBA)				
Physician/specialist	-4.88			-3.40
	[-11.61,1.85]			[-9.62,2.82]
Other/unskilled[§]	-0.81			11.27
	[-15.45,13.83]			[-2.15,24.69]
Don't Know/Couldn't distinguish	**-3.35***			-1.68
	**[-6.03,-0.67]**			[-4.34,0.97]
**Maternal PNC before discharge** (ref: No)				
Yes	**11.03****			-1.35
	**[7.74,14.33]**			[-6.58,3.89]
**Length of facility stay** (ref: <24h)				
≥24h	-1.26			-1.80
	[-3.72,1.20]			[-4.12,0.51]
**PNC counseling: danger signs** (ref: No)				
Yes	**7.81****			**2.99***
	**[5.46,10.16]**			**[0.13,5.84]**
**PNC counselling: family planning** (ref: No)				
Yes	**3.90****			0.27
	**[1.62,6.18]**			[-2.06,2.60]
**PNC: BP check** (ref: No)				
Yes	**8.54****			**3.97***
	**[5.96,11.12]**			**[0.61,7.34]**
**PNC: newborn check** (ref: No)				
Yes	**13.07****			**9.06****
	**[9.92,16.21]**			**[4.74,13.38]**
**PNC: newborn appointment** (ref: No)				
Yes	**8.28***			2.47
	**[1.56,14.99]**			[-3.72,8.66]
Observations (n)		401	377	371
AIC		3043.5	2883.0	2775.7

95% confidence intervals in brackets

* $p < 0.05$, ** $p < 0.01$

[§] small sample (≤10)

Supplemental tables 6A-C: Structural, intermediary and health systems determinants of PCMC domains by study site.

A. Lusaka

	Dignity & Respect (out of 18 points)	Communication & Autonomy (out of 27 points)	Supportive Care (out of 45 points)
STRUCTURAL DETERMINANTS			
**Education** (ref: primary or less)			
secondary or more	-0.39 [-1.08,0.31]	0.32 [-0.76,1.41]	-0.86 [-2.01,0.29]
**Employment** (ref: unemployed)			
Employed (public, private, self)	0.03 [-0.65,0.71]	**1.15*** **[0.09,2.20]**	0.16 [-0.95,1.27]
Informal/casual labor	-0.75 [-1.87,0.36]	0.01 [-1.73,1.74]	-0.21 [-2.04,1.63]
INTERMEDIARY DETERMINANTS			
**Age** (ref: 20-35 yrs)			
15-19 yrs	-0.56 [-1.65,0.54]	-1.35 [-3.05,0.35]	0.33 [-1.47,2.13]
35-49 yrs	0.01 [-1.12,1.13]	-0.20 [-1.95,1.55]	-0.49 [-2.34,1.35]
**Marital status** (ref: in union)			
Not in union	-0.22 [-1.05,0.62]	0.95 [-0.35,2.25]	1.08 [-0.30,2.45]
**Parity** (ref: 2-3 children)			
1	0.31 [-0.54,1.16]	0.10 [-1.22,1.43]	-0.99 [-2.39,0.41]
4+	-0.30 [-1.20,0.61]	-0.10 [-1.51,1.31]	0.18 [-1.30,1.67]
**Pregnancy complications** (ref: No)			
Yes	-0.17 [-1.24,0.90]	0.74 [-0.92,2.41]	-1.45 [-3.21,0.31]
**Miscarriage/Stillbirth history** (ref: No)			
Yes	-0.26 [-1.09,0.56]	-0.23 [-1.52,1.06]	-0.19 [-1.56,1.17]
**Number of ANC contacts** (ref: 1-3)			
0	-1.61 [-4.25,1.03]	1.00 [-3.12,5.11]	0.29 [-4.05,4.63]
4+	0.10 [-0.60,0.79]	0.13 [-0.96,1.22]	0.36 [-0.79,1.51]
**Place of ANC** (ref: Different facility/home/no ANC)			
Same facility as place of delivery	-0.39 [-1.06,0.28]	-0.53 [-1.58,0.52]	-0.58 [-1.69,0.53]

HEALTH SYSTEMS DETERMINANTS			
**Delivery facility type** (ref: Health center)			
Hospital	-0.11	0.24	-1.34
	[-1.44,1.22]	[-2.86,3.33]	[-3.94,1.26]
**Assistance during delivery** (ref: midwife/ nurse/TBA)			
Physician/specialist	0.42	**2.56****	1.07
	[-0.62,1.46]	**[0.94,4.18]**	[-0.64,2.78]
Other/unskilled	-0.55	1.49	-2.61
	[-3.33,2.24]	[-2.86,5.83]	[-7.20,1.97]
Don't Know/Could not distinguish	-0.03	-0.95	-2.09
	[-1.87,1.82]	[-3.82,1.93]	[-5.12,0.95]
**Maternal PNC before discharge** (ref: No)			
Yes	1.72	0.59	-0.55
	[-0.45,3.89]	[-2.79,3.96]	[-4.11,3.02]
**Length of facility stay** (ref: <24h)			
≥24h	-0.64	-0.70	-0.18
	[-1.35,0.08]	[-1.81,0.40]	[-1.35,1.00]
**PNC counseling: danger signs** (ref: No)			
Yes	**1.12****	**2.59****	**3.02****
	**[0.35,1.89]**	**[1.39,3.79]**	**[1.75,4.29]**
**PNC counseling: family planning** (ref: No)			
Yes	-0.34	0.03	-0.58
	[-1.05,0.37]	[-1.08,1.14]	[-1.75,0.59]
**PNC: BP check** (ref: No)			
Yes	0.58	-0.13	2.27
	[-1.11,2.27]	[-2.75,2.50]	[-0.50,5.05]
**PNC: newborn check** (ref: No)			
Yes	0.25	1.12	**3.41****
	[-1.10,1.59]	[-0.98,3.22]	**[1.20,5.63]**
**PNC: newborn appointment** (ref: No)			
Yes	0.94	-1.69	0.61
	[-0.77,2.65]	[-4.36,0.97]	[-2.20,3.43]
Observations	419	419	419

95% confidence intervals in brackets

* $p < 0.05$, ** $p < 0.01$

B. Nairobi

	Dignity & Respect (out of 18 points)	Communication & Autonomy (out of 27 points)	Supportive Care (out of 45 points)
STRUCTURAL DETERMINANTS			
**Education** (ref: primary or less)			
Secondary or more	-0.03 [-0.53,0.48]	0.08 [-0.79,0.95]	-0.36 [-1.28,0.56]
**Employment** (ref: unemployed)			
Employed (public, private, self)	0.52 [-0.04,1.07]	-0.37 [-1.33,0.58]	0.25 [-0.76,1.26]
Informal/casual labor	0.08 [-0.61,0.76]	-0.39 [-1.57,0.79]	0.69 [-0.56,1.94]
INTERMEDIARY DETERMINANTS			
**Age** (ref: 20-35 yrs)			
15-19 yrs	-0.22 [-1.21,0.78]	-0.31 [-2.03,1.41]	-0.25 [-2.08,1.57]
35-49 yrs	**0.91*** **[0.14,1.68]**	-0.25 [-1.58,1.08]	1.20 [-0.20,2.61]
**Marital status** (ref: in union)			
Not in union	-0.14 [-0.83,0.55]	**-1.24*** **[-2.43,-0.05]**	-1.16 [-2.42,0.11]
**Parity** (ref: 2-3 children)			
1	0.00 [-0.59,0.60]	**-1.02*** **[-2.03,-0.00]**	-0.26 [-1.33,0.82]
4+	0.24 [-0.53,1.01]	-0.28 [-1.61,1.05]	0.65 [-0.76,2.06]
**Pregnancy complications** (ref: No)			
Yes	0.00 [-0.65,0.66]	-0.29 [-1.42,0.84]	1.00 [-0.20,2.20]
**Miscarriage/Stillbirth history** (ref: No)			
Yes	-0.37 [-1.00,0.26]	-0.30 [-1.39,0.79]	-0.60 [-1.76,0.56]
**Number of ANC contacts** (ref: 1-3)			
0	-1.65 [-3.98,0.69]	0.70 [-3.34,4.74]	0.25 [-4.03,4.53]
4+	0.34 [-0.17,0.85]	0.86 [-0.02,1.74]	0.02 [-0.91,0.96]
**Place of ANC** (ref: Different facility/home/no ANC)			
Same facility as place of delivery	-0.22 [-0.75,0.32]	0.14 [-0.83,1.10]	-0.32 [-1.35,0.71]
HEALTH SYSTEMS DETERMINANTS			
**Delivery facility type** (ref: Health center)			
Hospital	-0.33 [-0.98,0.32]	**-1.90*** **[-3.63,-0.16]**	**-4.81**** **[-7.11,-2.51]**
**Delivery facility managing authority/ownership** (ref: Public)			
Private for profit	**1.26***	-0.45	1.67

	**[0.27,2.26]**	[-2.69,1.79]	[-1.10,4.44]
Private non-profit/faith-based	**0.89***	-0.58	0.67
	**[0.10,1.67]**	[-2.45,1.28]	[-1.71,3.04]
**Assistance during delivery** (ref: midwife /nurse /TBA)			
Physician/specialist	**1.03****	**2.03****	**1.74****
	**[0.55,1.52]**	**[1.16,2.90]**	**[0.81,2.66]**
Don't Know §	**2.16***	1.21	-1.37
	**[0.51,3.80]**	[-1.62,4.05]	[-4.37,1.63]
**Maternal PNC before discharge** (ref: No)			
Yes	0.98	0.24	1.89
	[-0.50,2.46]	[-2.32,2.81]	[-0.83,4.60]
**Length of facility stay** (ref: <24h)			
≥24h	0.38	-0.73	-0.48
	[-0.26,1.02]	[-1.86,0.40]	[-1.68,0.71]
**PNC counseling: danger signs** (ref: No)			
Yes	**0.65***	**2.88****	**1.08***
	**[0.12,1.18]**	**[1.94,3.81]**	**[0.09,2.08]**
**PNC counseling: family planning** (ref: No)			
Yes	-0.34	-0.21	-0.27
	[-0.84,0.17]	[-1.14,0.71]	[-1.26,0.72]
**PNC: BP check** (ref: No)			
Yes	0.27	0.95	1.02
	[-0.62,1.15]	[-0.57,2.47]	[-0.59,2.63]
**PNC: newborn check** (ref: No)			
Yes	**1.51****	**3.11****	**2.84****
	**[0.45,2.56]**	**[1.27,4.94]**	**[0.89,4.78]**
**PNC: newborn appointment** (ref: No)			
Yes	0.41	0.51	1.05
	[-0.87,1.69]	[-1.69,2.71]	[-1.28,3.38]
Observations	405	405	405

95% confidence intervals in brackets

* $p < 0.05$, ** $p < 0.01$

§ small sample (≤10)

C. Ouagadougou

	Dignity & Respect (out of 18 points)	Communication & Autonomy (out of 27 points)	Supportive Care (out of 45 points)
STRUCTURAL DETERMINANTS			
**Education** (ref: primary or less)			
Secondary or more	-0.15	0.22	0.57
	[-0.80,0.49]	[-0.71,1.15]	[-0.68,1.82]
**Employment** (ref: unemployed)			
Employed (public, private, self)	0.19	**1.94****	1.09
	[-0.66,1.05]	**[0.70,3.17]**	[-0.57,2.74]
Informal/casual labor	0.06	0.69	0.29
	[-0.58,0.70]	[-0.23,1.61]	[-0.94,1.52]
INTERMDIARY DETERMINANTS			
**Age** (ref: 20-35 yrs)			
15-19 yrs	0.84	1.01	0.66
	[-0.36,2.04]	[-0.72,2.74]	[-1.66,2.98]
35-49 yrs	0.34	0.33	0.84
	[-0.61,1.29]	[-1.04,1.70]	[-0.99,2.68]
**Marital status** (ref: in union)			
Not in union	-0.86	0.29	1.11
	[-3.13,1.41]	[-2.99,3.56]	[-3.27,5.49]
**Parity** (ref: 2-3 children)			
1	-0.43	-0.36	-0.42
	[-1.24,0.38]	[-1.54,0.81]	[-1.99,1.15]
4+	-0.27	0.12	-0.54
	[-1.05,0.51]	[-1.01,1.24]	[-2.04,0.96]
**Pregnancy complications** (ref: No)			
Yes	-0.04	0.75	-1.20
	[-1.06,0.98]	[-0.74,2.23]	[-3.23,0.82]
**Miscarriage/Stillbirth history** (ref: No)			
Yes	0.43	-0.37	1.12
	[-0.24,1.10]	[-1.33,0.60]	[-0.17,2.41]
**Number of ANC contacts** (ref: 1-3)			
0§	1.06	**3.07***	1.05
	[-0.81,2.94]	**[0.36,5.78]**	[-2.58,4.67]
4+	0.35	0.66	0.62
	[-0.27,0.98]	[-0.23,1.56]	[-0.57,1.82]
**Place of ANC** (ref: Different facility/home /no ANC)			
Same facility as place of delivery	0.04	0.56	0.35
	[-0.63,0.71]	[-0.42,1.53]	[-0.95,1.66]
HEALTH SYSTEMS DETERMINANTS			
**Delivery facility type** (ref: Health center)			
Hospital	-0.16	0.22	0.31
	[-1.19,0.87]	[-1.35,1.79]	[-1.99,2.61]
**Delivery facility managing authority/ownership** (ref: Public)			
Private for profit	0.84	**2.80***	**3.46***

	[-0.63,2.31]	**[0.57,5.04]**	**[0.23,6.69]**
Private non-profit/faith-based	0.23	0.63	2.24
	[-0.82,1.28]	[-0.97,2.23]	[-0.09,4.57]
**Assistance during delivery** (ref: midwife/ nurse/ TBA)			
Physician/specialist	-0.54	-2.01	-0.72
	[-2.33,1.26]	[-4.61,0.58]	[-4.19,2.75]
Other/unskilled §	**4.83***	1.90	4.81
	**[0.94,8.71]**	[-3.70,7.51]	[-2.70,12.31]
Don't Know	-0.48	-0.75	-0.38
	[-1.20,0.24]	[-1.81,0.32]	[-1.84,1.08]
**Maternal PNC before discharge** (ref: No)			
Yes	-0.39	-1.91	1.35
	[-1.91,1.12]	[-4.10,0.28]	[-1.58,4.28]
**Length of facility stay** (ref: <24h)			
≥24h	-0.44	-0.22	-1.01
	[-1.09,0.21]	[-1.17,0.73]	[-2.29,0.28]
**PNC counseling: danger signs** (ref: No)			
Yes	0.68	0.22	**2.05***
	[-0.13,1.49]	[-0.96,1.40]	**[0.46,3.64]**
**PNC counseling: family planning** (ref: No)			
Yes	-0.19	0.68	-0.27
	[-0.85,0.47]	[-0.28,1.64]	[-1.56,1.03]
**PNC: BP check** (ref: No)			
Yes	0.68	**2.57****	0.68
	[-0.29,1.65]	**[1.17,3.97]**	[-1.20,2.56]
**PNC: newborn check** (ref: No)			
Yes	**2.02****	**3.57****	**3.15***
	**[0.79,3.25]**	**[1.78,5.36]**	**[0.74,5.56]**
**PNC: newborn appointment** (ref: No)			
Yes	**1.81***	2.16	-1.31
	**[0.01,3.60]**	[-0.43,4.76]	[-4.78,2.15]
Observations	371	371	371

95% confidence intervals in brackets

* $p < 0.05$, ** $p < 0.01$

§ small sample (≤10)

Supplemental table 7 . PCMC supportive care item responses (% women) by delivery facility type in Nairobi (n=412)

Question	Response category	Health center/other (n=195) (%)	Hospital (n=217) (%)	Chi^2 p value
How did you feel about the amount of time you waited to receive care? Would you say it was:	Very long	51.8	30.4	<0.001
	Somewhat long	33.3	39.2	
	Somewhat short	10.8	23.0	
	Very short	4.1	7.4	
Did the doctors and nurses at the facility show concern for your feelings about your delivery?	No, never	0.5	8.3	<0.001
	Yes, a few times	7.7	31.8	
	Yes, most of the time	27.7	43.8	
	Yes, all the time	64.1	16.1	
Did the doctors, nurses, or other staff at the facility try to understand your anxieties?	No, never	4.1	12.0	<0.001
	Yes, a few times	4.1	21.2	
	Yes, most of the time	15.9	13.4	
	Yes, all the time	21.0	6.5	
	Not applicable	54.9	47	
When you needed help, did you feel the doctors, nurses, or other staff at the facility paid attention?	No,never	2.1	3.2	<0.001
	Yes, a few times	7.7	25.8	
	Yes, most of the time	27.2	50.2	
	Yes, all the time	63.1	20.7	
Do you feel the doctors or nurses did everything they could to help control your pain?	No,never	17.9	19.4	<0.001
	Yes, a few times	10.3	24.4	
	Yes, most of the time	22.1	35.5	
	Yes, all the time	49.7	20.7	

Were you allowed to have someone you wanted (outside of staff at the facility, such as family or friends) to stay with you during labor?	No, never	67.2	84.8	<0.001
	Yes, a few times	17.9	7.8	
	Yes, most of the time	2.1	2.3	
	Yes, all the time	8.2	0.9	
	I did not want someone to stay with me	4.6	4.1	
Were you allowed to have someone you wanted to stay with you during delivery?	No, never	86.2	90.3	0.305
	Yes, a few times	1.5	1.8	
	Yes, most of the time	0.5	0.5	
	Yes, all the time	3.1	0.5	
	I did not want someone to stay with me	8.7	6.9	
Did you feel the doctors, nurses, or other staff at the facility took good care of you, at the best of their ability?	No,never	0.5	2.8	<0.001
	Yes, a few times	1.5	8.8	
	Yes, most of the time	24.6	56.2	
	Yes, all the time	73.3	32.3	
Did you feel you could completely trust the doctors, nurses, or other staff at the facility with regards to your care?	No,never	1	0.9	<0.001
	Yes, a few times	3.1	6.5	
	Yes, most of the time	21.5	64.1	
	Yes, all the time	74.4	28.6	
Do you think there were enough health staff in the facility to care for you?	No,never	2.1	0.0	<0.001
	Yes, a few times	6.2	17.1	
	Yes, most of the time	19.5	50.2	
	Yes, all the time	72.3	32.7	

Thinking about the labor and postnatal wards, did you feel the health facility was crowded?	Yes, all the time	6.2	29.5	<0.001
	Yes, most of the time	3.6	11.5	
	Yes, a few times	9.2	14.7	
	No,never	81.0	44.2	
Thinking about the wards, washrooms, and the general environment of the health facility, would you say the facility was very clean, clean, dirty, or very dirty?	Very dirty	0.0	0.0	<0.001
	Dirty	0.0	5.5	
	Clean	23.6	69.1	
	Very clean	76.4	25.3	
Was there running water in the facility?	No,never	1.5	0.5	0.002
	Yes, a few times	2.1	1.4	
	Yes, most of the time	4.1	15.2	
	Yes, all the time	92.3	82.9	
Was there electricity in the facility?	No,never	0.5	0.0	0.005
	Yes, a few times	0.5	0.0	
	Yes, most of the time	3.6	12.4	
	Yes, all the time	95.4	87.6	
In general, did you feel safe in the health facility?	No,never	0.0	0.9	<0.001
	Yes, a few times	0.5	3.2	
	Yes, most of the time	6.2	47.5	
	Yes, all the time	93.3	48.4	
